# Cell migration and division in amoeboid-like fission yeast

**DOI:** 10.1242/bio.20136783

**Published:** 2013-12-17

**Authors:** Ignacio Flor-Parra, Manuel Bernal, Jacob Zhurinsky, Rafael R. Daga

**Affiliations:** Centro Andaluz de Biología del Desarrollo, Universidad Pablo de Olavide-Consejo Superior de Investigaciones Científicas, 41013 Sevilla, Spain; * Present address: Microbiology Department, Columbia University College of Physicians and Surgeons, New York, NY 10032, USA

**Keywords:** Cell migration, Cytokinesis, Fission yeast, Morphogenesis, Turgor pressure

## Abstract

Yeast cells are non-motile and are encased in a cell wall that supports high internal turgor pressure. The cell wall is also essential for cellular morphogenesis and cell division. Here, we report unexpected morphogenetic changes in a *Schizosaccharomyces pombe* mutant defective in cell wall biogenesis. These cells form dynamic cytoplasmic protrusions caused by internal turgor pressure and also exhibit amoeboid-like cell migration resulting from repeated protrusive cycles. The cytokinetic ring responsible for cell division in wild-type yeast often fails in these cells; however, they were still able to divide using a ring-independent alternative mechanism relying on extrusion of the cell body through a hole in the cell wall. This mechanism of cell division may resemble an ancestral mode of division in the absence of cytokinetic machinery. Our findings highlight how a single gene change can lead to the emergence of different modes of cell growth, migration and division.

## Introduction

Cellular morphogenesis requires polarization of the cytoskeleton and proper positioning of the cell division plane (Drubin and Nelson, 1996; Guertin et al., 2002). Fission yeast *Schizosaccharomyces pombe* are rod-shaped cells that grow by tip extension and divide by medial fission (Mitchison and Nurse, 1985). The spatial control of cell polarity and division in *S. pombe* makes this yeast a convenient model to study morphogenesis (Chang and Martin, 2009; Hayles and Nurse, 2001). Similar to other yeasts and fungi, *S. pombe* cells are surrounded by a cell wall, an extracellular matrix-like structure made of polysaccharides that allows the yeast cells to support the turgor pressure (Harold, 2002; Kopecká et al., 1995). Cell wall is a key regulator of cellular morphogenesis, and enzymatic removal of the cell wall results in rounded cells (protoplasts) unable to organize polarized growth zones and failing to divide (Osumi et al., 1989).

Free-living eukaryotic cells lacking a cell wall, such as amoebas, usually counteract turgor pressure by means of cortical actin cytoskeleton that generates a tension-resistant actomyosin cortex directly underlying the plasma membrane (Stockem et al., 1982). While such cells are unable to generate permanent rigid cell shapes, they, similarly to yeast and fungi that remodel the cell wall at the growth zones, rely on local weakening of the actomyosin cortex to allow cell expansion. In amoebas, this results in pseudopodium formation and movement (Webb and Horwitz, 2003) and in yeasts and fungi, produces polarized cell growth (Chang and Martin, 2009).

Actin polarization at the growth zones and proper function of the actomyosin division ring in *S. pombe* both rely on cell wall remodeling, resulting in tip growth and division septum assembly, respectively (Mulvihill et al., 2006; Santos et al., 2005). During tip growth, cell wall remodeling enzymes are transported in a polarized manner to the sites of growth to locally modify the cell wall and allow for its expansion partly driven by turgor pressure (Cortés et al., 2005; Cortés et al., 2002). The wall, in turn, is necessary for polarized growth zones to develop (Osumi et al., 1989). Thus, polarized cell growth, which involves addition of new membrane at growth sites, generates the characteristic cylindrical shape of fission yeast (Harold, 1990; Minc et al., 2009). Cell division in fission yeast, as in most eukaryotic cells, depends on an actomyosin ring (Marks et al., 1986). Ring contraction is coordinated with synthesis of new cell wall behind the closing ring, coupling actomyosin contraction to septum assembly. Thus, cell wall is involved in establishing and maintaining cell shape and also regulates cell division (Kobori et al., 1994; Madden and Snyder, 1998).

To probe the functions of the cell wall we analyzed cells lacking *pck2* gene (Toda et al., 1993). *pck2* encodes for one of the two protein kinase C homologues in *S. pombe* and is required for the activation of key enzymes that synthesize the β-1,3-glucan, a major structural component of the fission yeast cell wall that forms a fibrillary network responsible for its mechanical strength (Kobori et al., 1994; Kopecká et al., 1995; Osumi et al., 1998; Toda et al., 1993), and also regulates α-glucan biosynthesis (Calonge et al., 2000). We find that weak-walled *pck2*Δ cells are unable to establish and maintain cylindrical shape and, unexpectedly, form cytoplasmic protrusions apparently caused by cell wall rupture due to internal turgor pressure. Strikingly, multiple cycles of protrusion result in efficient cell migration. Moreover, protrusion events also allow cells to overcome failure of conventional cytokinesis and to divide using a novel mechanism relying on protrusion formation. Our data reveal how changes in a single gene can lead to the emergence of new morphogenetic properties and the generation of cellular movement in a non-motile yeast cell.

## Results

### Generation of fission yeast cells defective in cell wall biogenesis

When fission yeast cells are treated with lytic enzymes that digest the cell wall, they become rounded protoplasts (Osumi et al., 1989). Upon removal of the lytic enzymes, these protoplasts regenerate a new cell wall and re-form their rod shape. To interfere with cell wall biogenesis, we used *pck2Δ* cells. *pck2Δ* cells maintain functional cell wall during normal growth, but are unable to fully recover from protoplasting and only reassemble a weak or partial cell wall, which does not stain for β-1,3-glucans. These cells exhibit abnormal rounded cell shapes (Kobori et al., 1994) (see experimental design in supplementary material Fig. S1). When grown in osmotically stabilizing media, these *pck2Δ* cells after protoplast recovery (which we will refer to as “*RP-pck2Δ* cells”) epigenetically maintain abnormal morphology for many generations.

### *RP-pck2Δ* cells form cytoplasmic protrusions

**Fig. 1.**
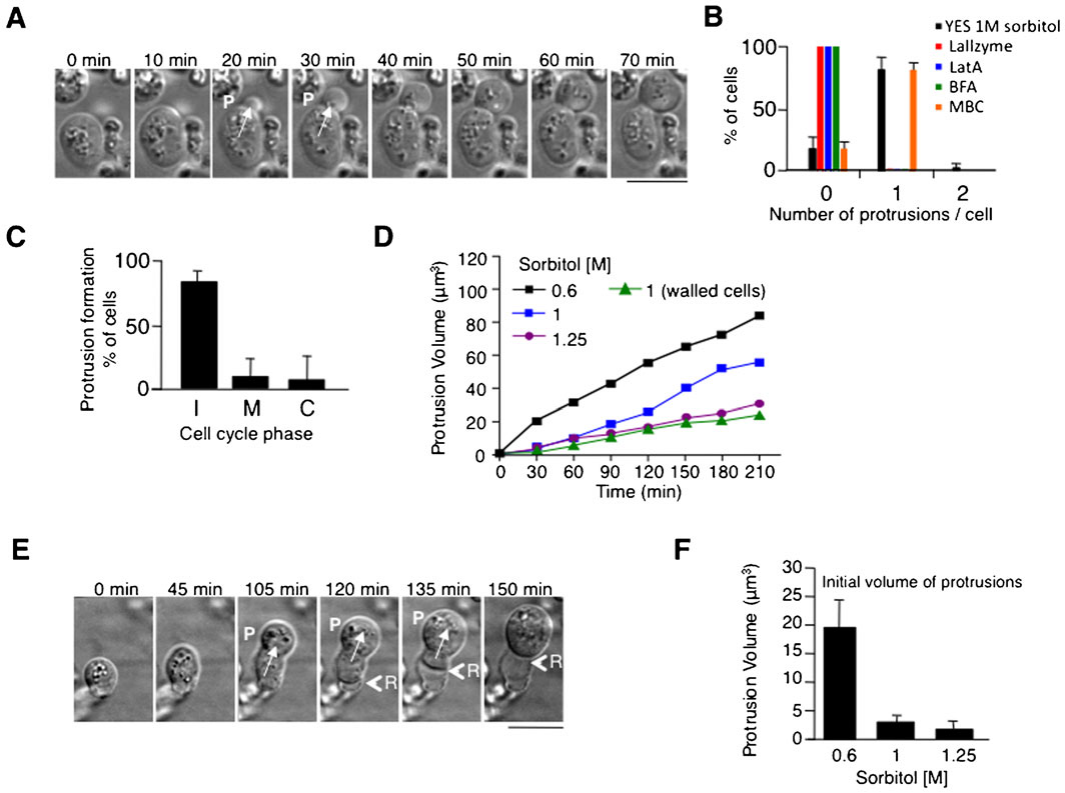
*pck2Δ* cells form cellular protrusions. (A) Time-lapse DIC images of *RP-pck2Δ* cells forming protrusions in 1 M Sorbitol. P marks appearance of a protrusion. (B) Percentage of cells with the indicated number of protrusions in the indicated conditions. (C) Cell cycle stage of cells initiating a protrusion. Frequency of protrusion appearance at different stages of the cell cycle (I interphase, M mitosis and C cytokinesis). (D) Protrusion volume increase in protruding *RP-pck2Δ*. Volume increase during growth of walled *pck2Δ* in 1 M Sorbitol is also shown. (E) Time-lapse DIC images of a protruding *RP-pck2Δ* cell showing the retraction (R) of the cell body as the protrusion expands. (F) Average protrusion volume in the first video frame where protrusion is visible in *RP-pck2Δ* cells at the indicated osmolarity. Scale bars: 5 µm.

To investigate how cell wall defects in *RP-pck2Δ* cells affect cell morphogenesis, we used time-lapse microscopy. We found that these cells often formed cytoplasmic protrusions, in which the cell appeared to slowly “flow out” from a hole in the cell wall. Protrusions were seen in 80% of cells (*n* = 50 cells) and their initiation required cells to reach a certain minimal volume (Fig. 1A; supplementary material Fig. S2A,B; Movie 1). Most cells (34/42 cells) formed a single protrusion at one time (Fig. 1B). The frequency of protrusion formation was between 0 to 2 events per cell cycle and they were observed at all cell cycle stages (Fig. 1C). Protrusions were not explained by cell growth, as their volume increase rate was faster than the rate of cell growth (Fig. 1D). Consistent with this, plasma membrane detached from the cell wall at the rear as the front of the cell protruded (Fig. 1E, time 120 min; supplementary material Movie 2). As fungal cells possess high internal turgor pressure (Bastmeyer et al., 2002; Harold, 2002; Minc et al., 2009), we tested whether this pressure drives protrusion events, by changing osmolarity of the medium. When we increased the effective turgor pressure by lowering sorbitol levels in the medium, the rate of protrusion growth increased (Fig. 1D,F). Protrusions did not depend on the nature of the osmostabilizer used, and also formed when sorbitol was replaced for polyethylene glycol or sucrose (data not shown). Similar protrusions were seen in wild-type fission yeast cells when the cell wall was digested with lytic enzymes, although the rates of protrusion in these cases were a magnitude faster (supplementary material Fig. S3A,B; Movie 3). It is likely that such increased rate would result from a total lack of cell wall at the plasma membrane of the protrusion, whereas in *RP-pck2Δ* cells some cell wall is likely present around the protrusion. Thus, our results suggest that protrusions are caused by internal turgor pressure forcing cellular contents out of a hole in the cell wall.

Protrusion formation required the presence of at least some cell wall, since cells in the continuous presence of the lytic enzyme exhibited no protrusions (Fig. 1B). F-actin disassembly by latrunculin A (LatA) (Ayscough et al., 1997) or inhibition of protein secretion with Brefeldin A (Klausner et al., 1992) completely abolished cell protrusions, while depolymerization of interphase microtubules had little effect (Fig. 1B). Thus, protrusion formation requires an intact actin cytoskeleton and/or membrane secretion. This could reflect a requirement for cell growth or for cell wall remodeling, which are dependent on actin and secretory pathways.

### *RP-pck2Δ* cells exhibit cell migration

**Fig. 2.**
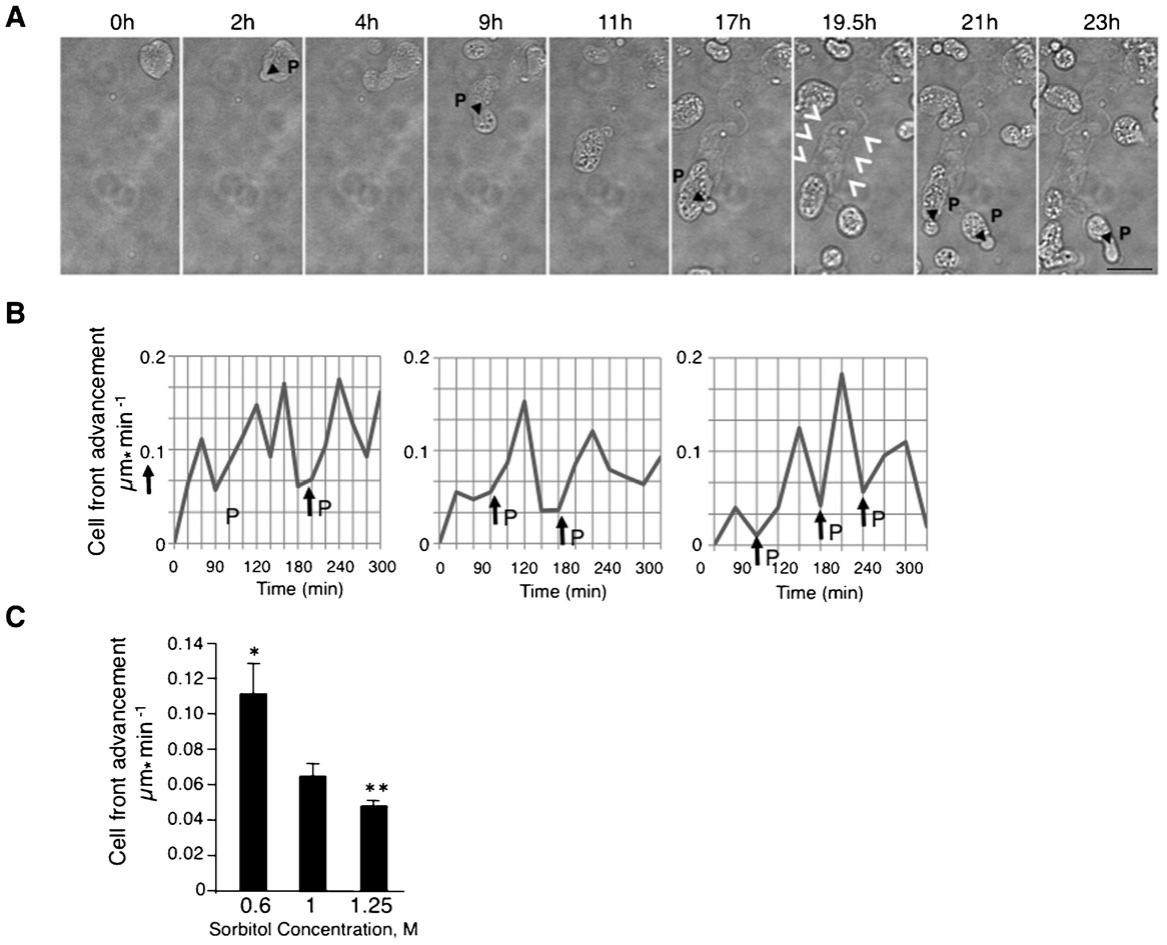
Cell migration of *RP-pck2Δ*. (A) Time-lapse bright-field images of protruding *RP-pck2Δ* cells. Arrows indicate appearance of cell protrusions (P). Arrowhead denotes the presence of a trail of cell material left behind during cell movement. Scale bar: 5 µm. (B) Increase in the rate of cell front advancement upon protrusion formation. Velocity averages over 30 minute timespan are shown during migration of protruding *pck2Δ* cells grown in 1 M Sorbitol. P indicates the protrusion appearance. (C) Average velocity of protruding *pck2Δ* cells grown at each of the indicated osmolarities. Error bars show SEM. The differences between rates of movement at different osmolarities (0.6 M vs 1 M and 1 M vs 1.25 M sorbitol) are statistically significant, indicated by asterisks (P<0.0001).

Strikingly, microscopic observation over many hours revealed amoeboid-like movement of *RP-pck2Δ* cells on agar pads (Fig. 2A; supplementary material Movie 4) (Webb and Horwitz, 2003; Yanai et al., 1996). Cells were observed to move across the agar for distances exceeding many cell lengths. Cell movement was also observed in cells attached to the glass surface in liquid medium, proving that movement is not a consequence of physical constraint imposed by the cover slip in agar pads (supplementary material Movie 5). The movement occurred at an average rate of 0.062±0.01 µm min^−1^ (in 1 M Sorbitol). The formation of a new protrusion was followed by an increase in velocity of cell movement (Fig. 2B). The migration rate was significantly higher (0.11±0.01 µm min^−1^) at low osmolarity (0.6 M sorbitol) and was reduced (0.046±0.002 µm min^−1^) at high osmolarity (1.25 M sorbitol) (Fig. 2C), suggesting that movement depends on turgor pressure. Like many motile cells, including amoebas and macrophages (Harshey, 2003; Kirfel et al., 2004; Uchida and Yumura, 2004), migrating *RP-pck2Δ* left a trail of materials behind them, presumably cell wall fragments (Fig. 2A, time 19.5 h, arrowheads). Thus, cell migration of *RP-pck2Δ* may be a result of repeated cycles of protrusion driven by internal turgor pressure, accompanied by weak cell wall rupture and repair.

### Actomyosin-independent division in protruding *RP-pck2Δ* cells

**Fig. 3.**
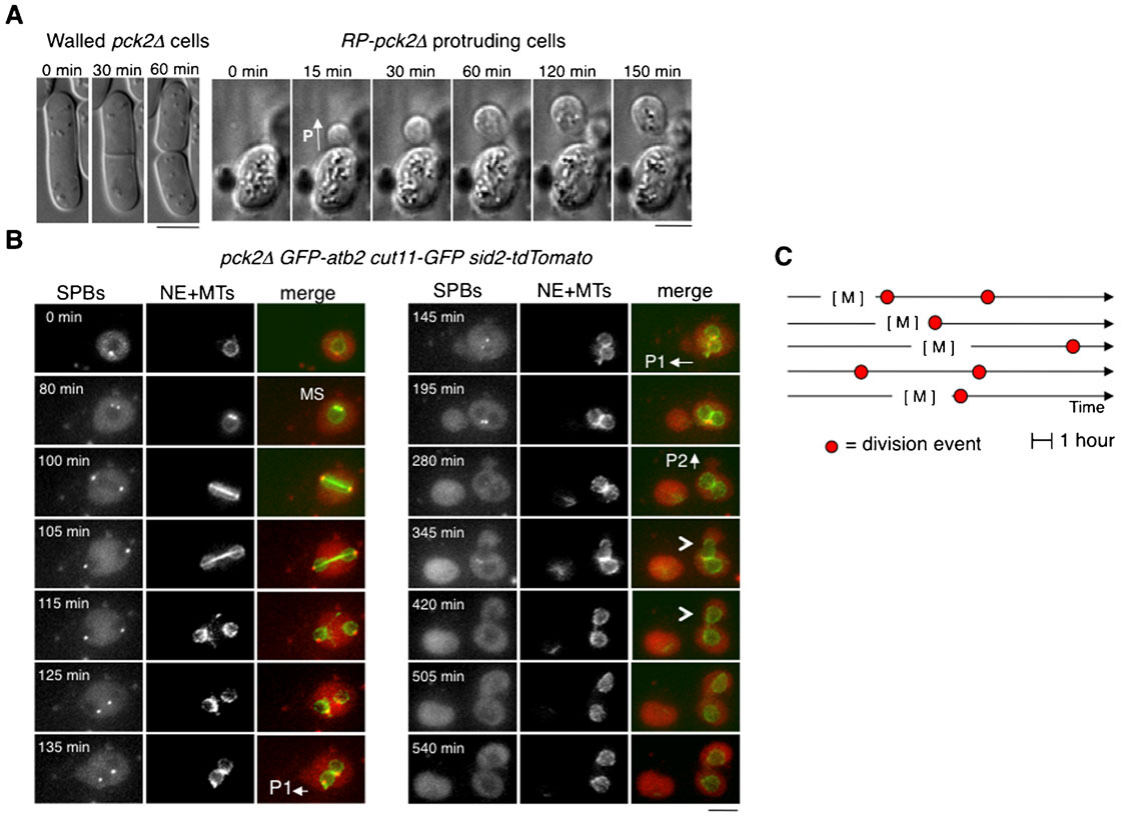
Cell division in *RP-pck2Δ* cells. (A) Time-lapse DIC images of a walled *pck2Δ* cell and bright field images of a protruding *pck2Δ* cell undergoing division. P denotes the formation of a cellular protrusion. (B) Lack of coordination between nuclear division and cell division in *RP-pck2Δ* cells. Protruding *pck2Δ* cell expressing GFP-atb2, cut11-GFP and Sid2-TOMATO as markers of the mitotic spindle (MS), nuclear envelop, and spindle pole body, respectively, were recorded in multiple focal planes every 5 minutes. Maximum z projections of representative time points are shown. Note mitotic spindle (MS) assembly at time 80 minutes, protrusion formation at 135 and 280 minutes (shown as P1 and P2), and cell separation events at 195 and 540 minutes. Arrowheads at time 345 and 420 minutes indicate the movement of one of the two nuclei present in the cell after the failure in conventional cytokinesis into the new cellular compartment as the protrusion grows. Note that the first protrusion is devoid of any nucleus representing the random nuclear segregation that occurs in protruding cells. Scale bars: 5 µm. (C) Cell cycle timing of seven representative division events corresponding to five independent cells. M indicates mitosis and red circles show the occurrence of cellular divisions.

During cell migration we observed that defective walled *RP-pck2Δ* exhibited an abnormal mode of cell division. In fission yeast, cell division normally involves the assembly and contraction of an actomyosin ring accompanied by the formation of the division cell wall septum (Krapp et al., 2004). Surprisingly, *RP-pck2Δ* cells frequently divided without the characteristic division septum of yeast cells (Fig. 3A,B, Fig. 2A; supplementary material Movie 6) in a manner uncoordinated with nuclear division (Fig. 3B,C). In contrast, mitotic spindles and nuclear division were apparently normal in *RP-pck2Δ* cells (Fig. 3B).

**Fig. 4.**
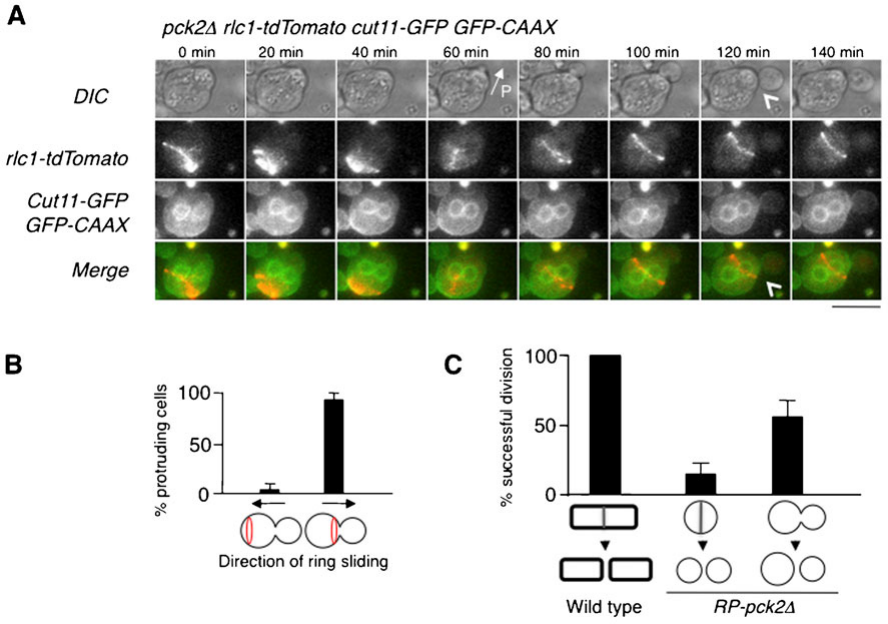
Actomyosin ring fails to divide most of *RP-pck2Δ* cells. (A) Cell separation independent of the actomyosin ring. Time lapse images of protruding *pck2Δ* cells expressing CAAX-GFP, Rlc1-tdTomato and Cut11-GFP as markers of the plasma membrane, actomyosin ring and nuclear envelope, respectively. Maximum z projections are shown. Scale bar: 5 µm. (B) Frequency of ring sliding towards the protrusion and away from it. (C) Percentage of walled and *RP-pck2Δ* cells undergoing successful divisions.

Consistent with a recent report (Mishra et al., 2012), the observation of actomyosin rings in these cells showed lateral ring sliding during assembly or contraction followed by ring collapse and division failure in most cells (67/78 cells) (supplementary material Movie 7). The sliding of these non-functional actomyosin rings was always directed towards the protrusion (Fig. 4A,B). After the first failure of cytokinesis, the ring reassembled again in 39% of cells (15/38 cells) and started to contract and slide with the same ineffective result (supplementary material Fig. S4A; Movie 8). The rate of ring contraction was similar to that observed in cells with an intact wall (supplementary material Fig. S4B).

**Fig. 5.**
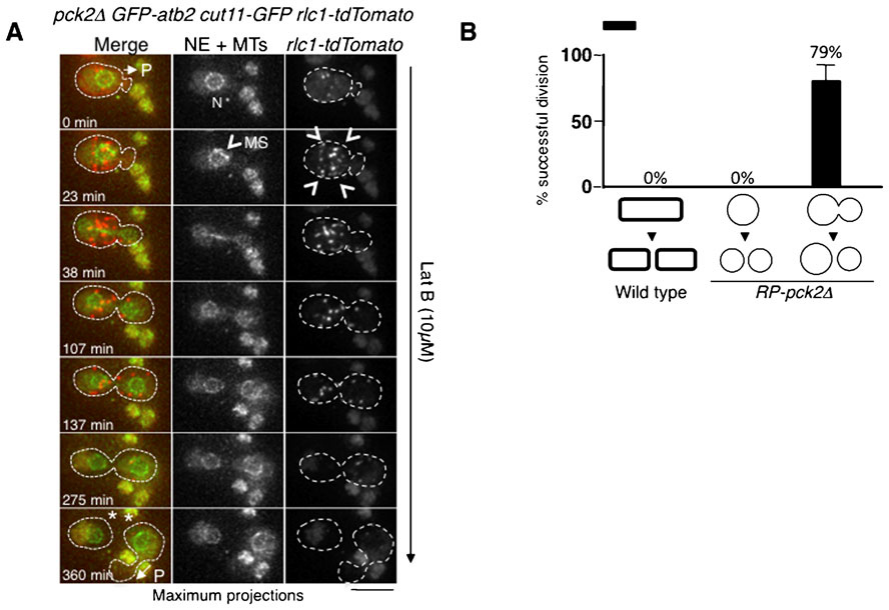
Cell division in the absence of actomyosin ring in *RP-pck2Δ* cells. (A) Time-lapse images of protruding *pck2Δ* cells expressing GFP-Atb2, Rlc1-tdTomato and Cut11-GFP as markers of the mitotic spindle, actomyosin ring and nuclear envelope, respectively. Cells were treated with 10 µM of LatB and recorded in multiple focal planes every 7.5 minutes. Maximum z-projections of representative time points are shown. P indicates the formation of a protrusion at time 0 and time 360 min, the asterisk denotes cell separation and N indicates the position of the nuclei. Arrowheads denote the myosin spots at the cell cortex indicative of an unassembled actomyosin ring. MS indicates the formation of the mitotic spindle at time 23 min. Dashed line highlights the cell border. Scale bar: 5 µm. (B) Percentage of *pck2Δ* and *RP-pck2Δ* cells undergoing successful divisions in the presence of 10 µM of LatB.

Surprisingly, most of the successful divisions in RP-*pck2Δ* occurred independently of the actomyosin ring. In 62% of cells (23/37) cell separation was observed to occur in the absence of detectable ring at the neck (Fig. 4C). In these cases, cells appeared to “pinch off” at the site of the initial protrusion (Fig. 4A, time 120 min; supplementary material Movie 9). To further test if this mode of division was independent of the actomyosin ring, we interfered with ring assembly. Since the presence of sorbitol, essential to maintain *RP*-*pck2Δ* cells, suppressed the phenotypes of the cytokinesis defective temperature sensitive mutants (data not shown), we decided instead to disrupt cytokinesis using Latrunculin B (LatB), a drug that prevents actomyosin ring formation by disrupting F-actin (Mulvihill and Hyams, 2002). Treatment of *RP-pck2Δ* cells with 10 µM LatB inhibited cytokinesis in all wild-type cells (*n* = 36 cells) but 79% of protruding cells (11/14 cells) were still able to divide in the presence of LatB, without any detectable actomyosin ring. We conclude that the actomyosin ring is not required for cell division in *RP-pck2Δ* cells (Fig. 5A,B; supplementary material Movie 10).

### Nuclear segregation in protruding *RP-pck2Δ* cells independently of mitotic spindle

**Fig. 6.**
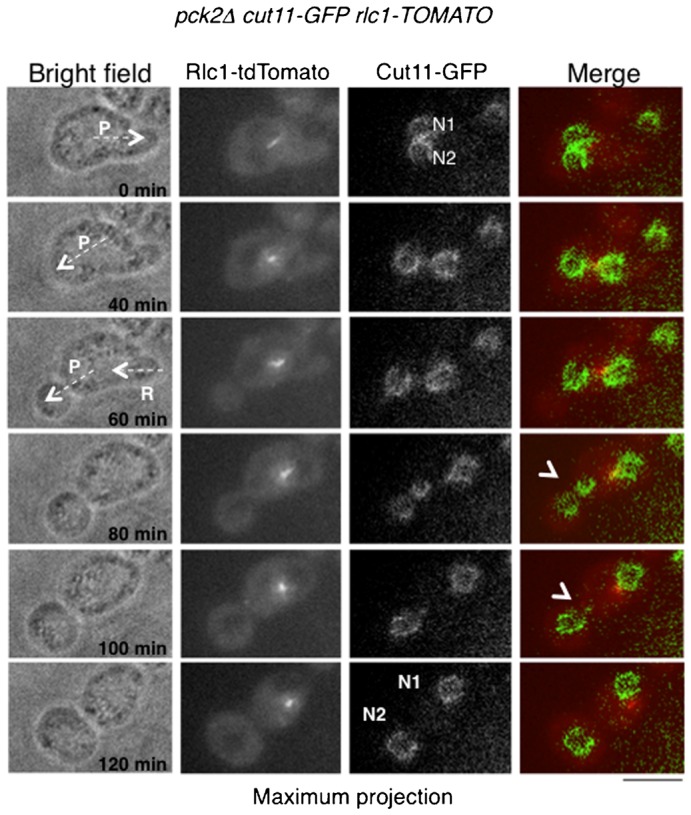
Nuclear segregation driven by protrusion formation. Protruding RP*-pck2Δ* cell expressing Rlc1-tdTomato and Cut11-GFP as markers of the actomyosin ring and nuclear envelope (NE) were recorded in multiple focal planes every 20 minutes. Maximum z projections of fluorescence and single sections of bright field images of representative time points are shown. P and arrow at time 0 min and 40 minutes indicate the formation of a protrusion. R at time 60 minutes denotes the retraction of a previously formed protrusion when the second one appeared. N1 and N2 show the two nuclei present in the cell. Arrowheads indicate deformation of the nucleus when it is passing through the neck toward the protrusion. Scale bar: 5 µm.

Many of cell divisions driven by protrusion formation were accompanied by nuclear segregation. When a protrusion was present at the time of mitotic spindle elongation, proper nuclear segregation occurred between the two compartments in all cases (12/12 cells). However, when protrusion appeared in cells that already contained two nuclei after a failure of cytokinesis, in 7/15 cells one of the two nuclei moved into the protrusion in the absence of the spindle and independently of mitosis. Nuclei appeared to be carried into the protrusion by cytoplasmic flow, though it is possible that endoplasmic reticulum based nuclear connections to the plasma membrane could also contribute to nuclear segregation (Fig. 6; supplementary material Movie 11). We also occasionally observed partial or total nuclear pinching when nuclei were crossing the narrow protrusion neck, similar to plasma membrane pinching (Fig. 6, time 80 min, arrowheads). The “pinching off” of the protrusion was independent of the presence of a nucleus in each compartment and 30% of pinching events (7/23 cells) resulted in the formation of anucleated compartments.

Thus, our data suggest that *RP-pck2Δ* cells can divide using an alternative mechanism that relies on cell protrusion through a small hole in the cell wall.

## Discussion

In this work we induced aspects of amoeboid-like cell migration and actomyosin ring-independent cytokinesis by weakening the cell wall in fission yeast cells. Cells with defective cell wall were generated by deleting the gene encoding the protein kinase C homologue, pck2, required for the synthesis of key components that provide the mechanical strength to the cell wall (Kobori et al., 1994; Kopecká et al., 1995). After enzymatic digestion of the cell wall, *pck2Δ* cells cannot fully regenerate and assemble a defective cell wall. In these cells, we observed protrusions resembling mammalian cell blebs. We interpret this as evidence of local rupture of the cell wall that leads to membrane being pushed through the hole. This interpretation is supported by the increase in the frequency of protrusion events and of the protrusion expansion rate at high intracellular turgor pressure. An intact actin cytoskeleton and/or membrane secretion is also required for protrusion formation.

Unexpectedly, repetitive cycles of protrusion formation resulted in cell migration. Cells moved over many cell lengths, usually in a single direction. A protrusion, or expansion of the cell forward, is always accompanied by detachment and retraction of the cytoplasm from the wall at the rear, resulting in an effective movement of the cell. Formation of a protrusion was frequently followed by an appearance of a new protrusion on its surface. This is likely a result of partial cell wall being formed at the surface of the expanding protrusion. It would explain the slow rate of protrusion expansion in *RP-pck2Δ* compared to that in the presence of cell wall digesting enzymes. Thus, migration in these cells may be a result of repeated cycles of protrusion driven by internal turgor pressure and accompanied by weak cell wall rupture and repair.

During amoeboid movement, local disruption of the actomyosin cortex results in cell expansion and the formation of pseudopodia. The amoeba cortex and the yeast cell wall are both locally modified or softened to allow cell expansion, which results in pseudopodia formation and in fungal growth, respectively (Chang and Martin, 2009; Harold, 2002; Stockem et al., 1982). In fungi, the overall cell volume increases as the cell grows while in amoebas, actomyosin contraction of the cortex at the rear leads to cell body translocation (Webb and Horwitz, 2003; Yoshida and Soldati, 2006). In *RP-pck2Δ*, we see a distinct phase of protrusion expansion, followed by the detachment of membrane at the rear. In animal cells, membrane detachment from the actin cortex, or local rupture of actin meshwork, induces the formation of cell protrusions or blebs (Charras et al., 2005; Diz-Muñoz et al., 2010; Tinevez et al., 2009). Blebbing induced by hydrostatic pressure has been proposed to be the driving force for movement of amoeboid (Langridge and Kay, 2006; Yanai et al., 1996; Yoshida and Soldati, 2006), embryonic cells (Blaser et al., 2006; Jaglarz and Howard, 1995) and also for tumor cell invasion (Charras and Paluch, 2008; Friedl and Wolf, 2003; Sahai and Marshall, 2003; Wolf et al., 2003).

Our findings demonstrate how a non-motile yeast cell can be transformed into an amoeboid form by weakening the cell wall, giving credence to the old idea that fungi may indeed be “amoebas in a tube” (Heath and Steinberg, 1999; Reinhardt, 1892).

It has previously been reported that cells deprived of cell wall fail to undergo cell division (Jochová et al., 1991). More recently, it has been shown that actomyosin rings in protoplasts slide sideways (Mishra et al., 2012). Consistent with this, our time-lapse experiments also show ring sliding in *RP-pck2Δ* cells, leading to the failure of septum assembly. Since round cells containing normal cell wall (orb6-25 and scd1Δ) are able to complete cell division and create a septum in most of the cases (Bernal et al., 2012; our unpublished data), it is possible that cell wall plays a role in anchoring the ring and preventing sliding. Importantly, this sliding was always directed towards the protrusion, suggesting that in addition to cell geometry (Mishra et al., 2012) and secretion (Pardo and Nurse, 2003), other factors such us flow of membrane and/or cytoplasm into the protrusion may contribute to directional ring sliding.

Surprisingly, after the failure of cytokinesis in *RP-pck2Δ* cells, the ring reassembled in a fraction of cells, similar to observations made in wild-type protoplasts suggesting the existence of a checkpoint-like control monitoring completion of cytokinesis (Mishra et al., 2012). This control could be novel or could partially overlap with mechanisms protecting the cell from partial damage to the ring (Le Goff et al., 1999; Liu et al., 2000).

Unexpectedly, in many cells cytoplasmic protrusion detached from the “mother cell”, generating two independent cellular compartments with no visible septum between them. Cell separation occurs at the narrow neck at the site of protrusion formation by a mechanism that is independent of the actomyosin ring and cell cycle stage. This conclusion is supported by our observations that cell separation occurs in cells with no visible actomyosin ring, in cells in which the ring is assembled in another part of the cell at the moment of cell separation, and in also cells treated with Latrunculin B, at a concentration that disassembles the ring. We speculate that membrane pinching and cell separation could result from cell membrane fusion, new membrane addition by vesicular transport, or from membrane breakage at the neck.

In the absence of nuclear segregation, cell separation would not be sufficient to overcome the failure of cytokinesis and to allow RP-*pck2Δ* cell proliferation. However, in many cells one of the nuclei moved into the protrusion, generating a functional daughter cell with normal ploidy. The nuclear segregation into the new compartment often occurred independently of mitotic spindle elongation and might be a consequence of cytoplasmic flow into the protrusion. We speculate that this division may be similar to the behavior of vesicles divided *in vitro* by extrusion when they are mechanically passed through a pore (Hanczyc et al., 2003; Mayer et al., 1986). This mode of cytokinesis by cell extrusion, independent of cytokinetic machinery, may resemble an ancestral mechanism of cell division in the absence of a contractile actomyosin ring. A similar mechanism of ftsZ-independent division, based on extrusion, has also been shown in bacteria lacking cell wall (Leaver et al., 2009). Interestingly, amoeboid slime mold cells are able to divide without myosin II or a discrete contractile ring (Neujahr et al., 1997; Zang et al., 1997).

Changes in cell wall may have underlain the evolution of different fungal forms. For instance, the pressure-driven protrusion events similar to the ones we describe could lead to the evolution of budding morphogenesis (Hartwell et al., 1974; Marchant and Smith, 1968). These findings potentially provide insights into the evolutionary relationships between walled cells such as fungi and more amoeboid forms, and how changes in cell wall metabolism and turgor pressure can lead to the evolution of different modes of cell growth, migration and division.

## Materials and Methods

### *S. pombe* strains, plasmids and media

The *S. pombe* strains used in this study are as follows: PPG42.10: h+ pck2:kan leu1-32 ura4-D18 (from Pilar Perez), RD716: h+ pck2:kan cut11-GFP:Ura4 Rlc1-TOMATO:Nat pREP3X-GFP-atb2, RD814: h+ pck2:kan cut11-GFP:ura4 Rlc1-TOMATO:Nat pREP3X-GFP-CAAX, RD696: h+ pck2:kan Rlc1-TOMATO:Nat pREP3X-GFP-atb2. Plasmid pREP3X-GFP-CAAX was constructed by cloning a PCR fragment encoding the last 19 amino acids from Cdc42p (Miller and Johnson, 1994) fused to the C-terminus of the green fluorescent protein (GFP). Cells were grown in YES or in MM-Leu when carrying plasmids and transferred to YES medium for 1 hour before forming protoplasts.

### Protoplast formation and protoplast recovery

To form protoplasts, cells were washed with SCS buffer and incubated with 0.1 g/ml Lallzyme MMX in SCS buffer (20 mM sodium citrate [pH 5.8] 0.6 M–1.5 M Sorbitol) until 100% of cells were converted to protoplasts. Time of incubation ranged from 8 minutes at 36°C to 12 min at 30°C. Protoplasts were harvested by centrifugation, inoculated into YES containing 1 M sorbitol (regeneration medium) (Kobori et al., 1989), and allowed to regenerate at 30°C for 4–6 hours.

### Microscopy and image analysis

Microscopy was performed at 25°C with either an upright wide-field fluorescence microscope or a delta vision confocal fluorescence microscope. Typically, confocal stacks were made of 12 z sections spaced by 0.5 µm taken with 0.5 s exposure, except Rlc1-TOMATO (0.1 s exposure). Images were acquired with MetaMorph software (Molecular Devices) and processed and analyzed with MetaMorph and Image J (http://imagej.nih.gov/ij). Movies of protoplast formation were taken at 30°C.

### Pharmacological inhibitors and drugs

Methyl-2-benzimidazole carbamate (MBC, Aldrich) was used at a final concentration of 50 µg/ml from a 100× stock solution made fresh in DMSO. Latrunculin A (LatA, Sigma) was used at a final concentration of 100 µM from a 200× stock in DMSO. Brefeldin A (BFA, Sigma) was used at a final concentration of 20 µg/ml from a 100× stock in ethanol. Latrunculin B (LatB, Sigma) was used at a final concentration of 10 µM from a 200× stock in DMSO. In the data presented, these drugs were added to the media 10 min prior to start of observation.

### Data quantification

The cell trajectories and velocity were measured by manually tracking the leading edge of each cell/protrusion using the Manual Tracking plugin of ImageJ. Protrusion volumes were approximated as spheric volumes based on the diameter of the protrusion. Divisions were recorded as successful when two compartments were generated from one. Rate of ring contraction of walled cells (*n* = 8) and RP-*pck2Δ* cells (*n* = 8) calculated by measuring ring diameter over time and calculating averages from the total contraction time.

### Statistical analyses

For determination of the statistical significance between two groups, the Student's t-test was used. Probability values (P<0.05) were considered to be statistically significant. Values depicted are means ± s.e.m.

## Supplementary Material

Supplementary Material

## References

[b1] Ayscough K. R. Stryker J. Pokala N. Sanders M. Crews P. Drubin D. G. (1997). High rates of actin filament turnover in budding yeast and roles for actin in establishment and maintenance of cell polarity revealed using the actin inhibitor latrunculin-A. J. Cell Biol. 137, 399–416 10.1083/jcb.137.2.399 9128251 PMC2139767

[b2] Bastmeyer M. Deising H. B. Bechinger C. (2002). Force exertion in fungal infection. Annu. Rev. Biophys. Biomol. Struct. 31, 321–341 10.1146/annurev.biophys.31.091701.170951 11988473

[b3] Bernal M. Sanchez-Romero M. A. Salas-Pino S. Daga R. R. (2012). Regulation of fission yeast morphogenesis by PP2A activator pta2. PLoS ONE 7, e32823 10.1371/journal.pone.0032823 22403715 PMC3293916

[b4] Blaser H. Reichman-Fried M. Castanon I. Dumstrei K. Marlow F. L. Kawakami K. Solnica-Krezel L. Heisenberg C. P. Raz E. (2006). Migration of zebrafish primordial germ cells: a role for myosin contraction and cytoplasmic flow. Dev. Cell 11, 613–627 10.1016/j.devcel.2006.09.023 17084355

[b5] Calonge T. M. Nakano K. Arellano M. Arai R. Katayama S. Toda T. Mabuchi I. Perez P. (2000). Schizosaccharomyces pombe rho2p GTPase regulates cell wall alpha-glucan biosynthesis through the protein kinase pck2p. Mol. Biol. Cell 11, 4393–4401 10.1091/mbc.11.12.4393 11102532 PMC15081

[b6] Chang F. Martin S. G. (2009). Shaping fission yeast with microtubules. Cold Spring Harb. Perspect. Biol. 1, a001347 10.1101/cshperspect.a001347 20066076 PMC2742080

[b7] Charras G. Paluch E. (2008). Blebs lead the way: how to migrate without lamellipodia. Nat. Rev. Mol. Cell Biol. 9, 730–736 10.1038/nrm2453 18628785

[b8] Charras G. T. Yarrow J. C. Horton M. A. Mahadevan L. Mitchison T. J. (2005). Non-equilibration of hydrostatic pressure in blebbing cells. Nature 435, 365–369 10.1038/nature03550 15902261 PMC1564437

[b9] Cortés J. C. Ishiguro J. Durán A. Ribas J. C. (2002). Localization of the (1,3)beta-D-glucan synthase catalytic subunit homologue Bgs1p/Cps1p from fission yeast suggests that it is involved in septation, polarized growth, mating, spore wall formation and spore germination. J. Cell Sci. 115, 4081–4096 10.1242/jcs.00085 12356913

[b10] Cortés J. C. Carnero E. Ishiguro J. Sánchez Y. Durán A. Ribas J. C. (2005). The novel fission yeast (1,3)beta-D-glucan synthase catalytic subunit Bgs4p is essential during both cytokinesis and polarized growth. J. Cell Sci. 118, 157–174 10.1242/jcs.01585 15615781

[b11] Diz-Muñoz A. Krieg M. Bergert M. Ibarlucea-Benitez I. Muller D. J. Paluch E. Heisenberg C. P. (2010). Control of directed cell migration in vivo by membrane-to-cortex attachment. PLoS Biol. 8, e1000544 10.1371/journal.pbio.1000544 21151339 PMC2994655

[b12] Drubin D. G. Nelson W. J. (1996). Origins of cell polarity. Cell 84, 335–344 10.1016/S0092-8674(00)81278-7 8608587

[b13] Friedl P. Wolf K. (2003). Tumour-cell invasion and migration: diversity and escape mechanisms. Nat. Rev. Cancer 3, 362–374 10.1038/nrc1075 12724734

[b14] Guertin D. A. Trautmann S. McCollum D. (2002). Cytokinesis in eukaryotes. Microbiol. Mol. Biol. Rev. 66, 155–178 10.1128/MMBR.66.2.155-178.2002 12040122 PMC120788

[b15] Hanczyc M. M. Fujikawa S. M. Szostak J. W. (2003). Experimental models of primitive cellular compartments: encapsulation, growth, and division. Science 302, 618–622 10.1126/science.1089904 14576428 PMC4484575

[b16] Harold F. M. (1990). To shape a cell: an inquiry into the causes of morphogenesis of microorganisms. Microbiol. Rev. 54, 381–431. 2128368 10.1128/mr.54.4.381-431.1990 PMC372787

[b17] Harold F. M. (2002). Force and compliance: rethinking morphogenesis in walled cells. Fungal Genet. Biol. 37, 271–282 10.1016/S1087-1845(02)00528-5 12431461

[b18] Harshey R. M. (2003). Bacterial motility on a surface: many ways to a common goal. Annu. Rev. Microbiol. 57, 249–273 10.1146/annurev.micro.57.030502.091014 14527279

[b19] Hartwell L. H. Culotti J. Pringle J. R. Reid B. J. (1974). Genetic control of the cell division cycle in yeast. Science 183, 46–51 10.1126/science.183.4120.46 4587263

[b20] Hayles J. Nurse P. (2001). A journey into space. Nat. Rev. Mol. Cell Biol. 2, 647–656 10.1038/35089520 11533722

[b21] Heath I. B. Steinberg G. (1999). Mechanisms of hyphal tip growth: tube dwelling amebae revisited. Fungal Genet. Biol. 28, 79–93 10.1006/fgbi.1999.1168 10587471

[b22] Jaglarz M. K. Howard K. R. (1995). The active migration of Drosophila primordial germ cells. Development 121, 3495–3503. 8582264 10.1242/dev.121.11.3495

[b23] Jochová J. Rupes I. Streiblová E. (1991). F-actin contractile rings in protoplasts of the yeast Schizosaccharomyces. Cell Biol. Int. Rep. 15, 607–610 10.1016/0309-1651(91)90007-6 1934083

[b24] Kirfel G. Rigort A. Borm B. Herzog V. (2004). Cell migration: mechanisms of rear detachment and the formation of migration tracks. Eur. J. Cell Biol. 83, 717–724 10.1078/0171-9335-00421 15679116

[b25] Klausner R. D. Donaldson J. G. Lippincott-Schwartz J. (1992). Brefeldin A: insights into the control of membrane traffic and organelle structure. J. Cell Biol. 116, 1071–1080 10.1083/jcb.116.5.1071 1740466 PMC2289364

[b601] Kobori H. Yamada N. Taki A. Osumi M. (1989). Actin is associated with the formation of the cell wall in reverting protoplasts of the fission yeast Schizosaccharomyces pombe. J. Cell Sci. 194, 635–646. 2630560 10.1242/jcs.94.4.635

[b26] Kobori H. Toda T. Yaguchi H. Toya M. Yanagida M. Osumi M. (1994). Fission yeast protein kinase C gene homologues are required for protoplast regeneration: a functional link between cell wall formation and cell shape control. J. Cell Sci. 107, 1131–1136. 7929623 10.1242/jcs.107.5.1131

[b27] Kopecká M. Fleet G. H. Phaff H. J. (1995). Ultrastructure of the cell wall of Schizosaccharomyces pombe following treatment with various glucanases. J. Struct. Biol. 114, 140–152 10.1006/jsbi.1995.1013 7612397

[b28] Krapp A. Gulli M. P. Simanis V. (2004). SIN and the art of splitting the fission yeast cell. Curr. Biol. 14, R722–R730 10.1016/j.cub.2004.08.049 15341766

[b29] Langridge P. D. Kay R. R. (2006). Blebbing of Dictyostelium cells in response to chemoattractant. Exp. Cell Res. 312, 2009–2017 10.1016/j.yexcr.2006.03.007 16624291

[b30] Le Goff X. Woollard A. Simanis V. (1999). Analysis of the cps1 gene provides evidence for a septation checkpoint in Schizosaccharomyces pombe. Mol. Gen. Genet. 262, 163–172 10.1007/s004380051071 10503548

[b31] Leaver M. Domínguez-Cuevas P. Coxhead J. M. Daniel R. A. Errington J. (2009). Life without a wall or division machine in Bacillus subtilis. Nature 457, 849–853 10.1038/nature07742 19212404

[b32] Liu J. Wang H. Balasubramanian M. K. (2000). A checkpoint that monitors cytokinesis in Schizosaccharomyces pombe. J. Cell Sci. 113, 1223–1230. 10704373 10.1242/jcs.113.7.1223

[b33] Madden K. Snyder M. (1998). Cell polarity and morphogenesis in budding yeast. Annu. Rev. Microbiol. 52, 687–744 10.1146/annurev.micro.52.1.687 9891811

[b34] Marchant R. Smith D. G. (1968). Bud formation in Saccharomyces cerevisiae and a comparison with the mechanism of cell division in other yeasts. J. Gen. Microbiol. 53, 163–169 10.1099/00221287-53-2-163 5721806

[b35] Marks J. Hagan I. M. Hyams J. S. (1986). Growth polarity and cytokinesis in fission yeast: the role of the cytoskeleton. J. Cell Sci Suppl. 5, 229–241 10.1242/jcs.1986.Supplement_5.15 3477553

[b36] Mayer L. D. Hope M. J. Cullis P. R. (1986). Vesicles of variable sizes produced by a rapid extrusion procedure. Biochim. Biophys. Acta 858, 161–168 10.1016/0005-2736(86)90302-0 3707960

[b600] Miller P. J. Johnson D. I. (1994). Cdc42p GTPase is involved in controlling polarized cell growth in Schizosaccharomyces pombe. Mol. Cell. Biol. 14, 1075–1083 10.1128/MCB.14.2.1075 8289788 PMC358463

[b37] Minc N. Boudaoud A. Chang F. (2009). Mechanical forces of fission yeast growth. Curr. Biol. 19, 1096–1101 10.1016/j.cub.2009.05.031 19500986 PMC2790036

[b38] Mishra M. Huang Y. Srivastava P. Srinivasan R. Sevugan M. Shlomovitz R. Gov N. Rao M. Balasubramanian M. (2012). Cylindrical cellular geometry ensures fidelity of division site placement in fission yeast. J. Cell Sci. 125, 3850–3857 10.1242/jcs.103788 22505610

[b39] Mitchison J. M. Nurse P. (1985). Growth in cell length in the fission yeast Schizosaccharomyces pombe. J. Cell Sci. 75, 357–376. 4044680 10.1242/jcs.75.1.357

[b40] Mulvihill D. P. Hyams J. S. (2002). Cytokinetic actomyosin ring formation and septation in fission yeast are dependent on the full recruitment of the polo-like kinase Plo1 to the spindle pole body and a functional spindle assembly checkpoint. J. Cell Sci. 115, 3575–3586 10.1242/jcs.00031 12186944

[b41] Mulvihill D. P. Edwards S. R. Hyams J. S. (2006). A critical role for the type V myosin, Myo52, in septum deposition and cell fission during cytokinesis in Schizosaccharomyces pombe. Cell Motil. Cytoskeleton 63, 149–161 10.1002/cm.20113 16421926

[b42] Neujahr R. Heizer C. Gerisch G. (1997). Myosin II-independent processes in mitotic cells of Dictyostelium discoideum: redistribution of the nuclei, re-arrangement of the actin system and formation of the cleavage furrow. J. Cell Sci. 110, 123–137. 9044043 10.1242/jcs.110.2.123

[b43] Osumi M. Yamada N. Kobori H. Taki A. Naito N. Baba M. Nagatani T. (1989). Cell wall formation in regenerating protoplasts of Schizosaccharomyces pombe: study by high resolution, low voltage scanning electron microscopy. J. Electron Microsc. (Tokyo) 38, 457–468. 2516868

[b44] Osumi M. Sato M. Ishijima S. A. Konomi M. Takagi T. Yaguchi H. (1998). Dynamics of cell wall formation in fission yeast, Schizosaccharomyces pombe. Fungal Genet. Biol. 24, 178–206 10.1006/fgbi.1998.1067 9742201

[b45] Pardo M. Nurse P. (2003). Equatorial retention of the contractile actin ring by microtubules during cytokinesis. Science 300, 1569–1574 10.1126/science.1084671 12791993

[b46] Reinhardt M. O. (1892). Das wachsthum der pilzhyphen. Jahrbücher für Wissenschaftliche Botanik 23, 479–566.

[b47] Sahai E. Marshall C. J. (2003). Differing modes of tumour cell invasion have distinct requirements for Rho/ROCK signalling and extracellular proteolysis. Nat. Cell Biol. 5, 711–719 10.1038/ncb1019 12844144

[b48] Santos B. Martín-Cuadrado A. B. Vázquez de Aldana C. R. del Rey F. Pérez P. (2005). Rho4 GTPase is involved in secretion of glucanases during fission yeast cytokinesis. Eukaryot. Cell 4, 1639–1645 10.1128/EC.4.10.1639-1645.2005 16215171 PMC1265894

[b49] Stockem W. Hoffmann H. U. Gawlitta W. (1982). Spatial organization and fine structure of the cortical filament layer in normal locomoting Amoeba proteus. Cell Tissue Res. 221, 505–519 10.1007/BF00215699 7198940

[b50] Tinevez J. Y. Schulze U. Salbreux G. Roensch J. Joanny J. F. Paluch E. (2009). Role of cortical tension in bleb growth. Proc. Natl. Acad. Sci. USA 106, 18581–18586 10.1073/pnas.0903353106 19846787 PMC2765453

[b51] Toda T. Shimanuki M. Yanagida M. (1993). Two novel protein kinase C-related genes of fission yeast are essential for cell viability and implicated in cell shape control. EMBO J. 12, 1987–1995. 8491190 10.1002/j.1460-2075.1993.tb05848.x PMC413420

[b52] Uchida K. S. Yumura S. (2004). Dynamics of novel feet of Dictyostelium cells during migration. J. Cell Sci. 117, 1443–1455 10.1242/jcs.01015 15020673

[b53] Webb D. J. Horwitz A. F. (2003). New dimensions in cell migration. Nat. Cell Biol. 5, 690–692 10.1038/ncb0803-690 12894172

[b54] Wolf K. Mazo I. Leung H. Engelke K. von Andrian U. H. Deryugina E. I. Strongin A. Y. Bröcker E. B. Friedl P. (2003). Compensation mechanism in tumor cell migration: mesenchymal-amoeboid transition after blocking of pericellular proteolysis. J. Cell Biol. 160, 267–277 10.1083/jcb.200209006 12527751 PMC2172637

[b55] Yanai M. Kenyon C. M. Butler J. P. Macklem P. T. Kelly S. M. (1996). Intracellular pressure is a motive force for cell motion in Amoeba proteus. Cell Motil. Cytoskeleton 33, 22–29 10.1002/(SICI)1097-0169(1996)33:1<22::AID-CM3>3.0.CO;2-K 8824731

[b56] Yoshida K. Soldati T. (2006). Dissection of amoeboid movement into two mechanically distinct modes. J. Cell Sci. 119, 3833–3844 10.1242/jcs.03152 16926192

[b57] Zang J. H. Cavet G. Sabry J. H. Wagner P. Moores S. L. Spudich J. A. (1997). On the role of myosin-II in cytokinesis: division of Dictyostelium cells under adhesive and nonadhesive conditions. Mol. Biol. Cell 8, 2617–2629 10.1091/mbc.8.12.2617 9398680 PMC25732

