# Supplementary material for: Cell migration and division in amoeboid-like fission yeast
Source: Biol Open. 2013 Dec 17;3(1):108–15. doi: 10.1242/bio.20136783 (PMC3892166; doi:10.1242/bio.20136783)
Supplement: Supplementary Material [file supp_3_1_108__index.html]

Cell migration and division in amoeboid-like fission yeast — Cell migration and division in amoeboid-like fission yeast — Supplementary Material 

# Cell migration and division in amoeboid-like fission yeast

## bio.20136783 Supplementary Material

**Files in this Data Supplement:**

- Supplementary Material - Ignacio Flor-Parra et al. doi: 10.1242/bio.20136783
- Movie 1 - **Movie 1. Protrusion formation in RP-*pck2Δ* cells.** Time-lapse bright-field images of RP-*pck2Δ* cells showing the appearance of cellular protrusions. Images were taken every of 30 minutes. The total time of the movie is 420 minutes.
- Movie 2 - **Movie 2. Protrusion formation and plasma membrane retraction of in *pck2Δ* cells.** Time-lapse DIC images of *pck2Δ* cells after protoplast recovery showing the retraction of the cell body at the rear as the protrusion expands. Images were taken every 15 minutes. The total time of the movie is 150 minutes.
- Movie 3 - **Movie 3. Protoplast formation in wild-type cells.** Time-lapse DIC images of wild-type cells treated with low dose of lytic enzyme (0.1 gr ml−1 Lallizime). Images were taken every minute. Total time of the movie is 11 minutes.
- Movie 4 - **Movie 4. Consecutive protrusions of RP-*pck2Δ* cells lead to cell movement.** Time-lapse bright field images of protruding *pck2Δ* cells observed on an agar pad were taken every 30 minutes. The total time of the movie is 23 hours.
- Movie 5 - **Movie 5. Protrusion-driven cell movement in liquid medium.** Time-lapse bright field images of protruding *pck2Δ* cells attached to the bottom of lectin-coated MatTek glass bottom culture dishes were taken every 10 minutes. The total time of the movie is 5 hours.
- Movie 6 - **Movie 6. Cell division in the absence of a division septum.** Time-lapse DIC images of dividing RP-*pck2Δ* cells. Note that two consecutive protrusions are formed and separated from the “mother” cell. Images were taken every 15 minutes. Total time of the movie is 150 minutes.
- Movie 7 - **Movie 7. Ring sliding in non-protruding *RP-pck2Δ* cells.** Maximum z projection of time-lapse images of *pck2Δ* cells expressing tubulin fused to GFP (GFP-atb2) to mark the mitotic spindle and myosin light chain Rlc1 fused to GFP (Rlc1-GFP) to mark the actomyosin ring. Images were taken every 10 minutes. The total time of the movie is 70 minutes.
- Movie 8 - **Movie 8. Repeated ring sliding and reassembly in *RP-pck2Δ* cells.** Maximum z projections of time-lapse images of *pck2Δ* cells expressing myosin light chain Rlc1 fused to GFP (Rlc1-GFP) to mark the actomyosin ring and cut11-GFP to mark the nuclear envelope. Images were taken in intervals of 5 minutes. The total time of the movie is 90 minutes.
- Movie 9 - **Movie 9. Ring sliding and cell division in protruding *RP-pck2Δ* cells.** Maximum z projection of time-lapse images of *pck2Δ* cells expressing the myosin light chain Rlc1 fused to GFP (Rlc1-GFP) to mark the actomyosin ring and CAAX-GFP to mark plasma membrane. Images were taken in multiple focal planes in intervals 10 minutes. Arrow denotes the formation of a protrusion. The total time of the movie is 140 minutes.
- Movie 10 - **Movie 10. Cell division in protruding *RP-pck2Δ* cells treated Latrunculin B.** Maximum z projection of time-lapse images of *pck2Δ* cells expressing the myosin light chain Rlc1 fused to GFP (Rlc1-GFP) to mark the actomyosin ring and tubulin fused to GFP (GFP-atb2) to mark the mitotic spindle. Cells were treated with 10 µM of Latrunculin B at time 0. Images were taken in multiple focal planes in intervals of 7.5 minutes. The total time of the movie is 360 minutes.
- Movie 11 - **Movie 11. Nuclear segregation in the absence of mitotic spindle in protruding *RP-pck2Δ* cells.** Time lapse images of *pck2Δ* cells expressing Rlc1-Tomato as actomyosin ring marker and Cut11-GFP as nuclear envelope marker, were recorded in multiple focal planes every 20 minutes. Maximum z projections are shown. The total time of the movie is 120 minutes.
