# Supplementary material for: Cell migration and division in amoeboid-like fission yeast
Source: Biol Open. 2013 Dec 17;3(1):108–15. doi: 10.1242/bio.20136783 (PMC3892166; doi:10.1242/bio.20136783)
Supplement: Supplementary Material [file supp_bio.20136783_bio.20136783-s1.pdf]

Supplementary Material

Ignacio Flor-Parra et al. doi: 10.1242/bio.20136783

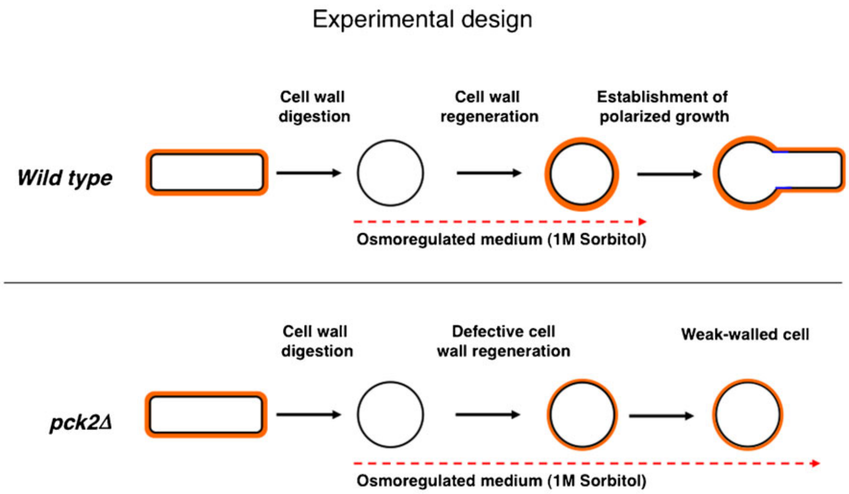

Fig. S1. Experimental design. The *S. pombe* cell wall was removed by enzymatic treatment with 0.1 g/ml of Lallzyme giving rise an unwalled cell (called protoplast). In osmoregulated medium, protoplasts regenerate the cell wall and initiate cell polarization to re-establish the fission yeast characteristic morphology. *pck2Δ* cells are unable to properly regenerate the cell wall, resulting in cells with a defective cell wall. The cell membrane is shown in blue and the cell wall in brown. For all experiments we used *pck2Δ* cells that have already regenerated the incomplete cell wall after recovery in YES+1 M Sorbitol for 4 hours (RP-*pck2Δ*). These cells were maintained in osmoregulated medium (1 M Sorbitol) to avoid cell lysis.

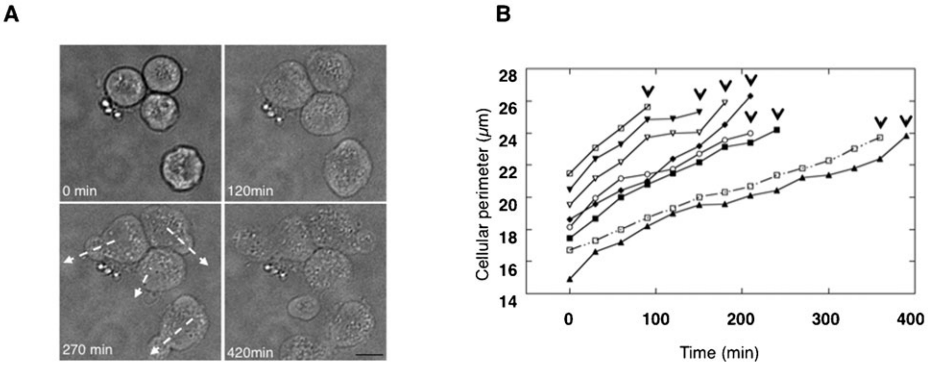

Fig. S2. *pck2Δ* cells recovered from protoplast form bleb-like protrusions after reaching a certain minimal volume. (A) Bright field images were recorded in single optical section every 30 minutes. Representative time points are shown. Arrows indicate protrusion formation. Scale bar: 5 µm. (B) Measurements of cellular perimeter over time as a proxy for cellular volume increase. Last point of each line represents the volume at which cells form a protrusion, indicated by an arrow.

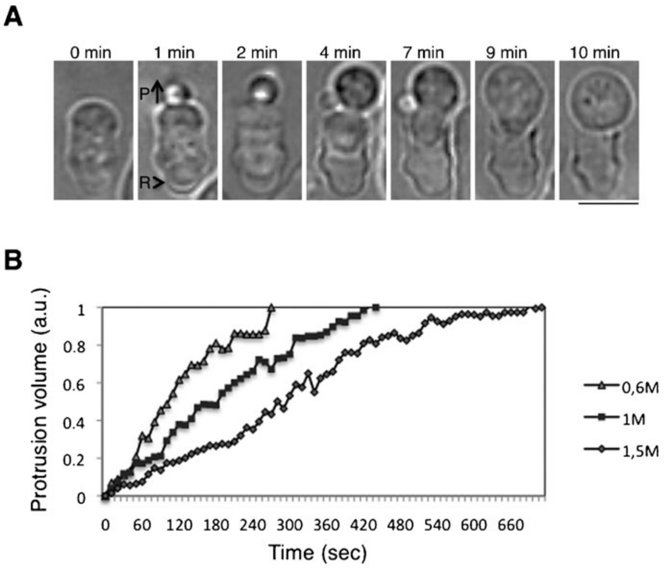

Fig. S3. Protrusion formation during protoplast generation in wild-type cells. (A) Bright field images were recorded in single optical section every minute. Representative time points are shown. Arrow indicates protrusion formation. Arrowheads and R indicate retraction. Scale bar: 5 µm.
(B) Osmolarity-dependent protrusion volume expansion in wild-type cells. Four cells were measured in each condition, final volumes were normalized and averages were calculated.

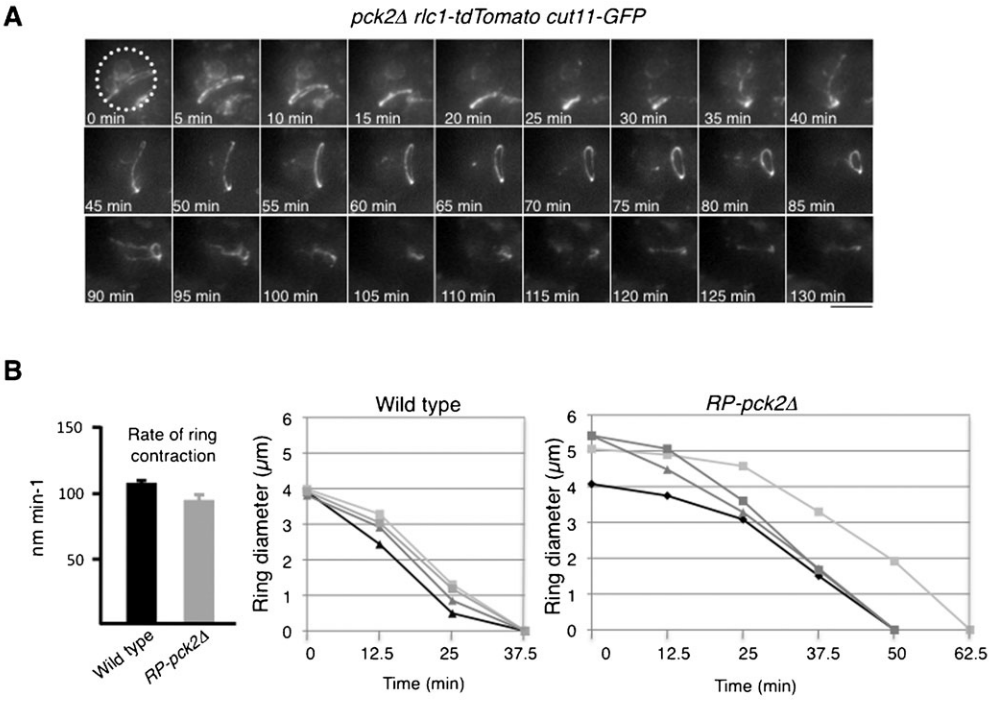

Fig. S4. Actomyosin ring behavior in *RP-pck2Δ*. (A) Repeated assembly and unproductive contraction of the actomyosin ring in *RP-pck2Δ*. *RP-pck2Δ* cells expressing Rlc1-tdTomato and Cut11-GFP to mark the actomyosin ring and nuclear envelope, respectively, were imaged in multiple focal planes every 5 minutes. Cell border is highlighted by dashed line at time 0. Scale bar: 5 µm. (B) Average rate of ring contraction in walled *pck2Δ* cells and in *pck2Δ* cells recovered from protoplast. Differences between rates of ring contraction in walled and weak-walled cells are not statistically significant. Four representative examples of ring diameter over time of both walled and weak-walled are shown.

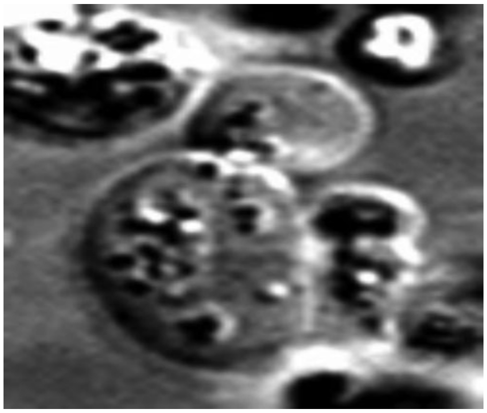

Movie 1. Protrusion formation in RP-*pck2Δ* cells. Time-lapse bright-field images of RP-*pck2Δ* cells showing the appearance of cellular protrusions. Images were taken every of 30 minutes. The total time of the movie is 420 minutes.

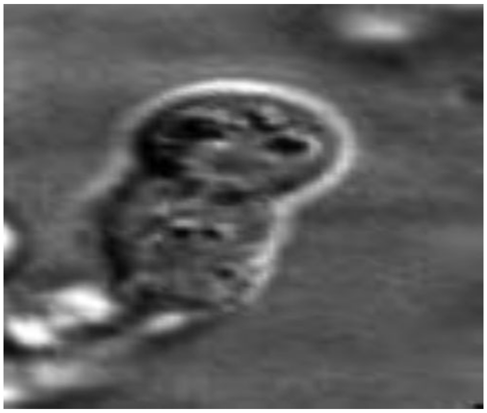

Movie 2. Protrusion formation and plasma membrane retraction of in *pck2Δ* cells. Time-lapse DIC images of *pck2Δ* cells after protoplast recovery showing the retraction of the cell body at the rear as the protrusion expands. Images were taken every 15 minutes. The total time of the movie is 150 minutes.

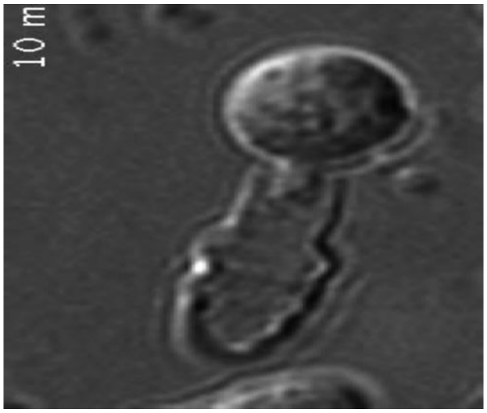

Movie 3. Protoplast formation in wild-type cells. Time-lapse DIC images of wild-type cells treated with low dose of lytic enzyme (0.1 gr ml^{-1} Lallizime). Images were taken every minute. Total time of the movie is 11 minutes.

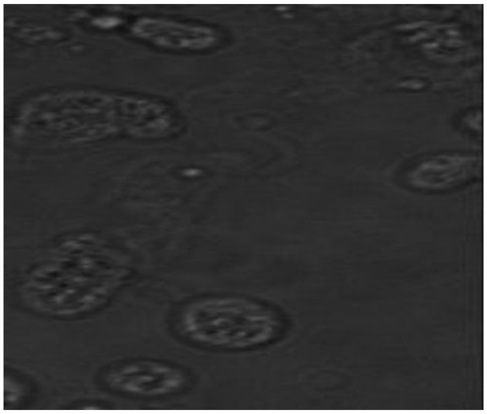

Movie 4. Consecutive protrusions of RP-*pck2Δ* cells lead to cell movement. Time-lapse bright field images of protruding *pck2Δ* cells observed on an agar pad were taken every 30 minutes. The total time of the movie is 23 hours.

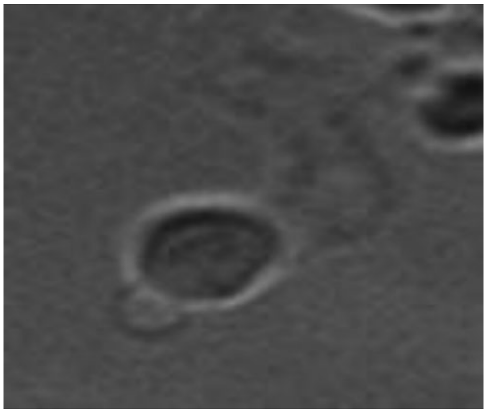

Movie 5. Protrusion-driven cell movement in liquid medium. Time-lapse bright field images of protruding *pck2Δ* cells attached to the bottom of lectin-coated MatTek glass bottom culture dishes were taken every 10 minutes. The total time of the movie is 5 hours.

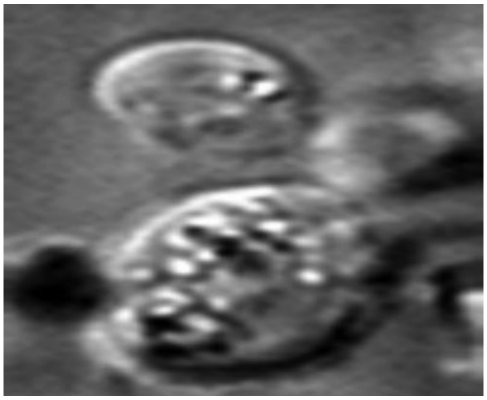

Movie 6. Cell division in the absence of a division septum. Time-lapse DIC images of dividing RP-*pck2Δ* cells. Note that two consecutive protrusions are formed and separated from the "mother" cell. Images were taken every 15 minutes. Total time of the movie is 150 minutes.

Biology Open

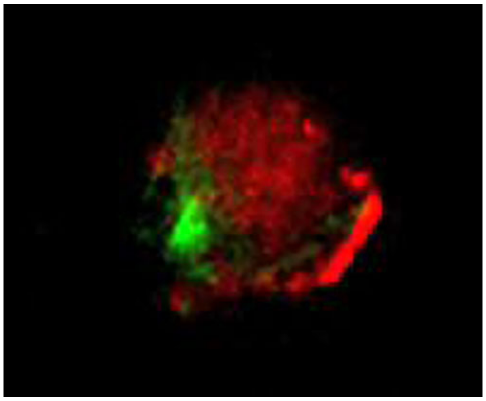

Movie 7. Ring sliding in non-protruding *RP-pck2Δ* cells. Maximum z projection of time-lapse images of *pck2Δ* cells expressing tubulin fused to GFP (GFP-atb2) to mark the mitotic spindle and myosin light chain Rlc1 fused to GFP (Rlc1-GFP) to mark the actomyosin ring. Images were taken every 10 minutes. The total time of the movie is 70 minutes.

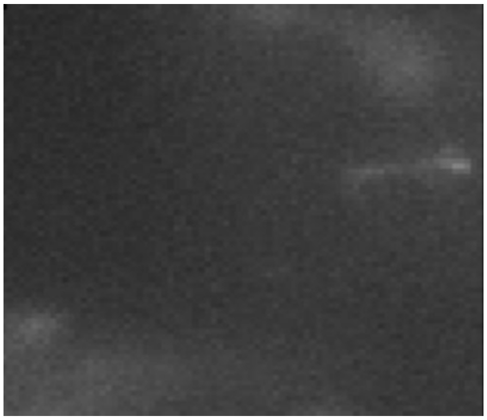

Movie 8. Repeated ring sliding and reassembly in *RP-pck2Δ* cells. Maximum z projections of time-lapse images of *pck2Δ* cells expressing myosin light chain Rlc1 fused to GFP (Rlc1-GFP) to mark the actomyosin ring and cut11-GFP to mark the nuclear envelope. Images were taken in intervals of 5 minutes. The total time of the movie is 90 minutes.

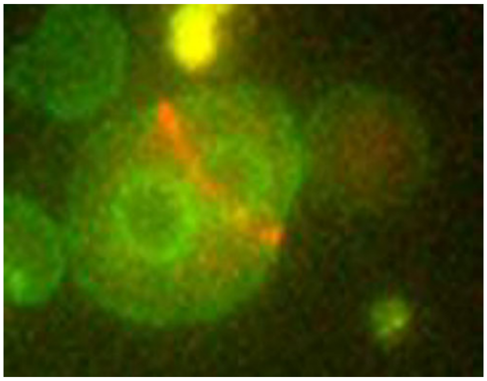

Movie 9. Ring sliding and cell division in protruding *RP-pck2Δ* cells. Maximum z projection of time-lapse images of *pck2Δ* cells expressing the myosin light chain Rlc1 fused to GFP (Rlc1-GFP) to mark the actomyosin ring and CAAX-GFP to mark plasma membrane. Images were taken in multiple focal planes in intervals 10 minutes. Arrow denotes the formation of a protrusion. The total time of the movie is 140 minutes.

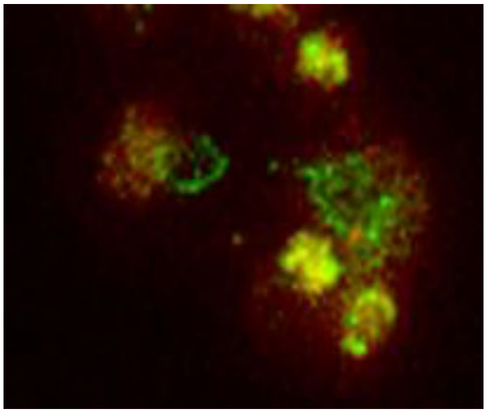

Movie 10. Cell division in protruding *RP-pck2Δ* cells treated Latrunculin B. Maximum z projection of time-lapse images of *pck2Δ* cells expressing the myosin light chain Rlc1 fused to GFP (Rlc1-GFP) to mark the actomyosin ring and tubulin fused to GFP (GFP-atb2) to mark the mitotic spindle. Cells were treated with 10 µM of Latrunculin B at time 0. Images were taken in multiple focal planes in intervals of 7.5 minutes. The total time of the movie is 360 minutes.

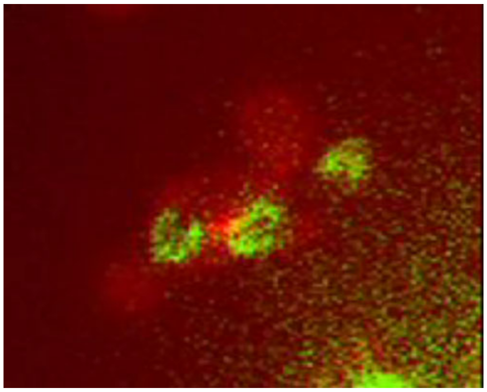

Movie 11. Nuclear segregation in the absence of mitotic spindle in protruding *RP-pck2Δ* cells. Time lapse images of *pck2Δ* cells expressing Rlc1-Tomato as actomyosin ring marker and Cut11-GFP as nuclear envelope marker, were recorded in multiple focal planes every 20 minutes. Maximum z projections are shown. The total time of the movie is 120 minutes.
